# Validation of an evaluation instrument for responders in tactical casualty care simulations*

**DOI:** 10.1590/1518-8345.3052.3251

**Published:** 2020-04-17

**Authors:** Maria Del Carmen Usero-Pérez, Maria Lourdes Jiménez-Rodríguez, Alexandra González-Aguña, Valentín González-Alonso, Luis Orbañanos-Peiro, Jose María Santamaría-García, Jorge Luís Gómez-González

**Affiliations:** 1 Academia Central Defensa, Escuela Militar De Sanidad, Madrid, Madrid, Spain.; 2 Universidad de Alcalá de Henares, Facultad de Ciencias de la Computación, Alcalá de Henares, Madrid, Madrid, Spain.

**Keywords:** Rubric, Validation, Emergency Services, Simulation, Terrorism, Training, Rubrica, Validação, Serviços de Emergência, Simulação, Terrorismo, Treinamento, Rúbrica, Validación, Servicios de Emergencias, Simulación, Terrorismo, Formación

## Abstract

**Objective:**

to construct and validate a tool for the evaluation of responders in tactical casualty care simulations.

**Method:**

three rubrics for the application of a tourniquet, an emergency bandage and haemostatic agents recommended by the Hartford Consensus were developed and validated. Validity and reliability were studied. Validation was performed by 4 experts in the field and 36 nursing participants who were selected through convenience sampling. Three rubrics with 8 items were evaluated (except for the application of an emergency bandage, for which 7 items were evaluated). Each simulation was evaluated by 3 experts.

**Results:**

an excellent score was obtained for the correlation index for the 3 simulations and 2 levels that were evaluated (competent and expert). The mean score for the application of a tourniquet was 0.897, the mean score for the application of an emergency bandage was 0.982, and the mean score for the application of topical haemostats was 0.805.

**Conclusion:**

this instrument for the evaluation of nurses in tactical casualty care simulations is considered useful, valid and reliable for training in a prehospital setting for both professionals who lack experience in tactical casualty care and those who are considered to be experts.

## Abstract (pt)

**Objetivo:**

construir e validar um instrumento de avaliação da prática, por meio da simulação, nos cuidados de saúde estratégicos.

**Método:**

três instrumentos para práticas de avaliação de aplicação do torniquete, bandagem de emergência e agente hemostático recomendados pelo Consenso de Hartford foram desenvolvidos e validados. A validade e a confiabilidade foram estudadas. A validação foi realizada por quatro especialistas da área e trinta e seis enfermeiros participantes selecionados por amostragem por conveniência. Três instrumentos de avaliação com 8 itens foram avaliados (com exceção da bandagem de emergência, que tinha 7 itens para avaliar). Cada prática foi avaliada por três especialistas.

**Resultados:**

uma pontuação excelente foi obtida no cálculo do índice de correlação para as três práticas e nos dois níveis avaliados (competente e especialista). A pontuação média para a aplicação do torniquete foi de 0,897, para o curativo de emergência foi de 0,982 e para a aplicação de agentes hemostáticos tópicos foi de 0,805.

**Conclusão:**

este instrumento de avaliação da prática por meio de simulação nos cuidados de saúde estratégicos é considerado útil, válido e confiável para o treinamento no contexto pré-hospitalar tanto dos profissionais que não possuem experiência nos cuidados estratégicos quanto nos considerados peritos.

## Abstract (es)

**Objetivo:**

construir y validar un instrumento de evaluación de la práctica, mediante simulación, en la atención sanitaria táctica.

**Método:**

se construyeron y validaron tres rúbricas de las prácticas de aplicación del torniquete, vendaje de emergencia y agente hemostático recomendadas por el Consenso Hartford. Se estudió la validez y fiabilidad. La validación se realizó por cuatro expertos en la materia y treinta y seis participantes enfermeros que fueron la muestra de conveniencia. Se evaluaron tres rúbricas con 8 ítems (excepto para el vendaje de emergencia que fueron 7 ítems a evaluar). Cada práctica fue evaluada por tres expertos.

**Resultados:**

se ha obtenido una excelente puntuación en el cálculo del índice de correlación para las tres prácticas y en los dos niveles evaluados (competente y experto). La puntuación media para la rúbrica de aplicación del torniquete fue de 0.897, la del vendaje de emergencia 0.982 y para la aplicación de hemostáticos tópicos 0.805.

**Conclusión:**

este instrumento de evaluación de la práctica mediante simulación en la atención sanitaria se considera útil, válido y fiable para la formación en el entorno prehospitalario tanto de profesionales que carecen de experiencia en atención táctica como de aquellos considerados como expertos.

## Introduction

Prehospital care includes a wide range of health interventions, and the environment in which it is performed often complicates care because of the danger of staying on the scene (explosions, traffic accidents, extreme temperatures, etc.); therefore, the assessment must be done quickly^(^ 1 ^)^.

The rapid assessment of dangerous environments is one of the challenges in emergency care during terrorist attacks. Recently, terrorist attacks in different parts of the world have involved the use of explosives, vehicle collisions, and active shooters, demonstrating the need to develop medical care systems in “unsafe” environments. An incident with an active shooter is defined as “an individual actively engaged in killing or attempting to kill people in a confined and populated area; in most cases, active shooters use firearms(s), and there is no pattern or method to their selection of victims”^(^ 2 ^)^.

Prehospital health care must adapt its procedures so that interventions in this environment are performed with maximum safety, both for the patient and for the responding personnel. Based on this adequacy requirement, in 2013, recommendations of the Hartford Consensus^(^ 3 ^)^were published. These procedures originated from the experience gained by military casualty care in recent years through the care of those wounded by firearms or explosives in military conflicts in areas such as Iraq or Afghanistan. During combat, caring for the wounded is carried out following the Tactical Combat Casualty Care (TCCC) recommendations, in which special importance is given to the treatment of external bleeding, airway obstruction and tension pneumothorax. Supported by this experience, the recommendations of the Hartford Consensus are applied to civilian prehospital care in risky environments such as terrorist attacks, emphasizing the importance of the rapid control of external bleeding of victims by the first responder (security forces, health personnel) as well as the rapid transfer of the wounded to a safe area and evacuation by medical services to a centre used to receive definitive treatment. The recommendations provide guidance to security forces for basic tactical casualty care^(^ 3 ^)^ (for our study, we define tactical casualty care as that carried out in risky environments with scarce resources, such as care provided during terrorist attacks). The recommendations of the Consensus establish 3 action zones as a function of environmental threat (hot, warm and cold zones), and tactical considerations are emphasized as determinants in prehospital care^(^ 4 ^)^.

The recommendations of the Hartford Consensus should be adapted to the different health systems of each country, especially training in the application of tourniquets, haemostatic dressings and compressive bandages^(^ 3 ^)^.

Tourniquets are recommended for external bleeding from extremities when the control of bleeding is ineffective or impossible through direct pressure. Haemostatic agents should be used in combination with direct pressure to control major bleeding. Compressive bandages can be used to control external bleeding^(^ 5 ^-^ 7 ^)^. Nurses play fundamental roles in these types of incidents^(^ 8 ^)^, both as first responders, adapting their care to an environment with special characteristics, and in developing collaborations in medical care and in joint learning with other professionals. Nursing is positioned as a connecting link between medical care personnel^(^ 9 ^)^.

One of the main points advocated is the development of training strategies for both first responders and professionals (health and non-health) involved in incidents in emergency environments to improve the survival of victims. Caring for victims is a responsibility shared by both the security forces involved and by the medical teams that care for them^(^ 3 ^)^.

Prehospital providers should be prepared to address situations of extreme urgency, but despite this premise, these situations represent a relatively low percentage of total care provided by these providers^(^ 10 ^)^. In light of this knowledge, there is a need to provide experience in the care of seriously injured patients through other means, such as simulations^(^ 11 ^)^. For health care needed due to terrorist acts, improved training in controlling bleeding for the different responding teams, in providing early and effective triage and in simulations of incident management for multiple victims is necessary^(^ 12 ^)^.

Well-trained responders involved in the control of external bleeding increases the chances of the survival of victims of a terrorist attack^(^ 13 ^)^.

In the same way that clinical staff perform evidence-based care, educators should follow the same pattern. Too much interest in learning from training results can lead to neglecting the development of valid instruments for obtaining the results. If the information for medical care training in emergency environments is obtained through reliable and valid tests, policies and programmes can be developed to improve the training. Validation is necessary to ensure that the results obtained are correct and to obtain assessment instruments that adapt to the needs of instructors^(^ 14 ^)^.

The absence of similar studies and the need for the evaluation of training lead to the objective of this study: to construct and validate a tool for the evaluation of responders in tactical casualty care simulations.

## Method

A validation study of an assessment instrument was designed for the following tactical casualty care simulations: application of a tourniquet, application of haemostatic dressings, application of an emergency bandage.

These tasks were chosen because they are basic techniques that should be known by all first responders^(^ 7 ^)^.

The study consisted of 3 phases.

*Phase 1* consisted of the development of an assessment instrument for each of the 3 tasks. These instruments were obtained in 3 steps.

To compile the different items that make up each instrument, information was obtained from the observation of cognitive tasks by 2 experts in the subject and was supported with reference manuals.

Once the rubrics were created, estimation of the construct validity was performed using expert-based judgement and the use of statistical methods derived from the application of the measurement instrument, such as the content validity index (CVI)^(^ 15 ^)^.

The experts who were selected to establish construct validity were 4 instructors from the Military Medical Care School (Escuela Militar de Sanidad, EMISAN) with recognized experience in the field (more than 3 years of teaching experience and conducting advanced training courses, with publications and teacher accreditations, in the subject in question). The judges were selected by convenience sampling.

Based on the levels of expertise in the Benner model^(^ 16 ^)^ (beginner, advanced beginner, competent, efficient and expert), 3 levels of training were proposed that corresponded to first responders (beginner), medical personnel with no experience in emergencies (competent) and medical staff with experience in prehospital care (expert). Thus, these 3 levels include practically all professionals who must be trained for interventions in this emergency environment. For this study, due to access to the sample, only validation was carried out at the competent and expert levels. The experts (selected by convenience) had to assess the inclusion of each item in the instruments for the 3 levels of knowledge proposed (beginner, competent and expert).

In addition to proposing inclusion for each of the levels, the experts assessed each item individually to determine whether it was appropriate and if it should be retained in the final version. Each item was assigned a score based on 3 possibilities: essential to evaluate the construct, useful but expendable, or unnecessary.

Finally, the CVI was calculated for the instrument as a whole. Because there were 4 experts, for an item to be included, a minimum score of 0.9 was needed^(^ 15 ^)^.

For those items that did not reach the minimum score, a new review was performed, obtaining a definitive version after reaching total agreement among the instructors who performed the assessment.

*Phase 2*: To prepare the evaluation, a preliminary meeting was held to resolve item disagreements between evaluators. A pilot study was conducted prior to the evaluation study using 15 nursing students from EMISAN (professionals with a bachelor’s degree or diploma in nursing carrying out technical training prior to committing to a military career) considered as a “competent” group. During simulations, each item was evaluated independently by the 3 instructors. This test served to familiarize instructors with the evaluation instrument and to modify items if necessary.
*Phase 3*: To calculate reliability, Cronbach’s alpha and the correlation coefficient index (CCI) were determined.

A Cronbach’s alpha value above 0.70 is considered acceptable. CCI values below 0.40 represent low reliability, between 0.40 and 0.75 represent fair to good reliability, and above 0.75 represent excellent reliability^(^ 17 ^)^.

Agreement was calculated using the CCI during the simulation of 3 tasks, such as hot zone application of a tourniquet and application of an emergency bandage and haemostatic dressings in a non-threatening environment.

The sample was defined for the 2 levels that were to be evaluated: competent (health professionals without experience in prehospital care) and expert (health professionals with experience in prehospital care). For the present study, a sample of 26 nursing students (competent level) from EMISAN were selected by convenience sampling, in addition to 10 students who participated in advanced training courses at the school (expert level). The latter had experience in international missions and provided similar training to these simulations. Each of the students (competent and expert) was evaluated by the 3 instructors, with a grade of YES/NO (depending on whether they completed the evaluated task). If the simulation was completed correctly, the maximum score was 10. Some of the items are tasks whose performance is considered essential.

The test was developed as follows:

For the competent level, the participants were provided with 2 hours of training in which the procedures for correct application of each device were explained. Two days later and in an EMISAN classroom, the 3 tasks were performed by each student.

Three stations were created, with each station evaluated by 3 observers.

For the application of a tourniquet, a Tourniquet in Emergency (TIE™) model was used; this task is habitually practised at EMISAN. The tourniquet was self-applied to the arm. A box of gloves was provided.

For the application of an emergency bandage, the participant decided between an arm or leg bandage. A box of gloves was provided.

For the application of a haemostatic dressing, Celox Gauze™ (gauze bandage) was used, in addition to a box of gloves and a low-cost thigh wound simulator.

For the expert level, no prior training was given because they were considered experts in the field. The simulation was conducted in the same way as in the previous group.

To carry out this study, permission was requested from the Central Defense Academy for the collection and analysis of the results. All participants gave their consent to participate in the study.

Calculations were performed using International Business Machines Statistical Package for the Social Sciences (IBM SPSS) 24.

## Results

*Phase 1 Results*: After calculating the CVI for the rubric for the application of a tourniquet, 3 items were extracted that did not reach the minimum desired score (0.9). After a second meeting in which a final drafting was carried out, the rubric to validate was unanimously accepted (Tables 1 and 2).
  **Table 1:** Results of Phase 1 of the study in which the validity of the evaluation instrument for the application of a tourniquet was evaluated. Madrid, CM, Spain, 2018

1^st^ Version	Panel of experts				CVI*	2^nd^ Version
	1	2	3	4		
**Application of a tourniquet in a hot zone**						
Instructs the injured person to control the bleeding by self-care, if able to.	E^†^	E	E	B^‡^	0	Rejected
Applies measures to protect against biological agents.	E	B	E	E	0.5	Rejected
Positions the tourniquet correctly on the arm 4 fingers from the shoulder joint, leaving space for the application of a second tourniquet if necessary.	E	E	E	E	1	Remains
Passes the end of the strap through the inner slit of the buckle.	B	E	E	E	0.5	Rejected
Fastens the tourniquet around the arm using the velcro strap.	E	E	E	E	1	Remains
Twists the tensioning rod until bleeding stops.	E	E	E	E	1	Remains
Secures the tensioning rod into both of its 2 safety tabs.	E	E	E	E	1	Remains
Places the velcro safety strap over the tensioning rod to secure it and prevent it from accidentally releasing.	E	E	E	E	1	Remains
Passes the remaining strap through the first buckle slit.	E	E	E	E	1	Remains
Folds the excess band over itself and tightens it with your hand.	E	E	E	E	1	Remains
Writes 'T' on the front and the starting time of ischaemia.	E	E	E	E	1	Remains

* CVI = Content Validity Index. Student level:

† E = Expert,

‡ B = Beginner
  **Table 2:** Results of Phase 1 in which the validity of the evaluation instrument for the application of haemostats and emergency bandage was evaluated. Madrid, CM, Spain, 2018

1^st^ Version	Panel of experts				CVI*	2^nd^ Version
	1	2	3	4		
**Application of a haemostatic dressing**						
Checks safety in the area.	E^†^	E	B^‡^	B	0	Rejected
Puts/has on nitrile gloves.	E	E	E	E	1	Remains
If there is a tourniquet, re-evaluates the previously placed tourniquet. Exposes the wound, and determines if a tourniquet is necessary.	E	E	E	E	1	Remains
Evaluates the wound to determine if a tourniquet is necessary.	E	E	E	E	1	Remains
Removes excess blood from the wound with a compress.	E	E	E	E	1	Remains
Applies haemostatic agent directly to the bleeding site.	E	E	E	E	1	Remains
Applies 3 minutes of direct pressure on the wound.	E	E	E	E	1	Remains
Provides coverage by placing a haemostatic emergency bandage over the wound.	E	E	E	E	1	Remains
Re-evaluates bleeding from the wound.	E	E	E	E	1	Remains
**Application of an emergency bandage**						
Puts/has on nitrile gloves.	E	E	E	E	1	Remains
Prepares the material.	B	B	B	I	-1	Rejected
If the wound has a tourniquet, it is loosened without removing it to check the site of bleeding.	B	B	E	B	-0.5	Rejected
Applies the sterile dressing over the wound, and wraps it around until reaching the plastic pressure clamp.	E	E	E	E	1	Remains
Tucks the bandage inside the pressure clamp, and reverses the direction of the bandage.	E	E	E	E	1	Remains
Exerts pressure on the pressure clamp, which should be positioned on top of the sterile dressing over the wound, and on each turn, pressure is applied until the entire bandage is used.	E	E	E	E	1	Remains
Secures the bandage with the clamp.	E	E	E	E	1	Remains
Evaluates distal pulses and sensitivity.	E	E	E	E	1	Remains
Monitors the onset/increase in bleeding.	E	E	E	E	1	Remains

* CVI = Content Validity Index; Student level:

† B = Beginner;

‡ E = Expert

For the rubric for emergency bandaging, the same procedure was performed: an item was removed that did not achieve the minimum score, and after applying the same test on volunteers, 7 items that constituted the rubric were considered for inclusion.

The evaluation of the application of haemostatic agents led to the removal of 2 items that did not reach the minimum score for inclusion, in addition to an editorial review; therefore, 8 items were used to assess this task.

During Phase 2 of the study, no changes were made to the rubric.
*Results of Phase 3*: The sociodemographic data for the participants are provided in Table 3.
  **Table 3:** Sociodemographic data for the participants. Madrid, CM, Spain, 2018

Variables	Pilot Study	Competent Level	Expert Level
Total	15	26	10
Sex			
Male	40%(6)	50% (13)	40% (4)
Female	60%(9)	50% (13)	60%(6)
Mean age	25.40	26.7	39.0 years
Time in AF* (mean)	New	New	10.9 (years)
Teaching experience	no	No	yes
Number of international missions in which they participated (mean)	0	0.03	3.6

* AF = Armed Forces

The CCI was adequate for most items, as shown in Tables 4 and 5.

**Table 4:** Correlation coefficient index (CCI*) results for the application of a tourniquet (competent and expert levels). Madrid, CM, Spain, 2018

Item	Competent			Expert		
	CCI*	*p*	Cronbach's alpha	CCI*	*p*	Cronbach's alpha
**Application of a tourniquet**						
Places the tourniquet correctly on the arm 4 fingers from the shoulder joint, leaving space for the application of a second tourniquet if necessary.	0.633	≤0.001	0.705	1.000^†^	≤0.001	1.000^†^
Fastens the tourniquet around the arm using the velcro strap.	1.000^‡^			1.000^‡^		
Twists the tensioning rod until bleeding stops.	1.000^‡^			1.000^‡^		
Secures the tensioning rod into both of its 2 safety tabs.	0.650	≤0.001	0.644	1.000^‡^		
Place the velcro safety strap over the tensioning rod to secure it and prevent it from being accidentally released.	0.800	≤0.001	0.794	1.000^†^	≤0.001	1.000^†^
Passes the remaining band through the first buckle slit.	0.767	≤0.001	0.762	1.000 ^†^	≤0.001	1.000^†^
Folds the excess band over itself and tightens it by hand.	1.000^†^	≤0.001	1.000^†^	1.000^†^	≤0.001	1.000^†^
Writes a "T" on the front and the starting time of ischaemia.	1.000^†^	≤0.001	1.000^†^	1.000^†^	≤0.001	1.000^†^
Points	0.724	≤0.001	0.748	0.990	≤0.001	0.992

* CCI = Correlation Coefficient Index;

† Agreement is 100%; the 3 instructors make the same assessment for each student: yes or no;

‡ Agreement is 100% for all YES or NO results of the 3 instructors for all students

**Table 5:** Correlation coefficient index (CCI*) results for the application of a haemostatic agent and an emergency bandage (competent and expert levels). Madrid, CM, Spain, 2018

Item	Competent			Expert		
	CCI*	*p*	α^†^	CCI*	*p*	α^†^
**Application of a topical haemostatic agent**						
Puts/has on nitrile gloves.	0.844	≤0.001	0.846	1.000^‡^	≤0.001	1.000^‡^
If there is a tourniquet, re-evaluates the previously placed tourniquet. Exposes the wound, and determines if the tourniquet is necessary.	0.810	≤0.001	0.816	0.609	0.074	0.636
Evaluates the wound to determine if a tourniquet is necessary.	0.731	≤0.001	0.765	0.372	0.262	0.356
Removes excess blood from the wound with the aid of a compress.	1.000	≤0.001	1.000^‡^	1.000^‡^	≤0.001	1.000^‡^
Applies haemostatic agent directly to the bleeding site.	1.000^§^			1.000^§^		
Applies 3 minutes of direct pressure to the wound.	1.000^§^			0.780	0.017	0.780
Covers the wound with a haemostatic emergency bandage.	0.921	≤0.001	0.924	1.000^§^		
Re-evaluates bleeding from the wound.	0.866	≤0.001	0.875	0.597	0.064	0.656
**Points**	0.842	≤0.001	0.838	0.905	≤0.001	0.909
**Application of an emergency bandage**						
Has/puts on nitrile gloves.	0.898	≤0.001	0.898	1.000^‡^	≤0.001	1.000^‡^
Applies the sterile dressing over the wound, wraps it around until reaching the plastic pressure clamp.	1.000^§^			1.000^§^		
Inserts the bandage into the pressure clamp, and reverses direction.	1.000^§^			1.000^§^		
Exerts pressure on the pressure clamp, which is positioned over the sterile dressing on the wound, and for each turn, pressure is applied until the entire bandage is used.	1.000^§^			1.000^§^		
Secures the bandage with the clamp.	1.000^§^			1.000^§^		
Evaluates distal pulses and sensitivity.	1.000^‡^	≤0.001	1.000^‡^	1.000^‡^	≤0.001	1.000^‡^
Monitors the onset/increase in bleeding.	0.982	≤0.001	0.982	1.000^‡^	≤0.001	1.000^‡^
**Points**	0.980	≤0.001	0.981	1.000^‡^	≤0.001	1.000^‡^

* CCI = Correlation Coefficient Index;

† Cronbach's alpha;

‡ Agreement is 100%; the 3 instructors agree on the assessment (YES or NO) for the item for all students evaluated;

§ Agreement is 100%; the 3 instructors agree on the assessment (YES or NO) for each student

The data obtained for items 2, 3 and 8 for the application of haemostats did not reach the minimum recommended score. In a subsequent analysis, combining the competent and expert level groups as a single sample, the score obtained indicated good reliability; the lowest values were obtained for item 3, with a CCI of 0.424, a significance of 0.047 and a Cronbach’s alpha of 0.437. This calculation was performed with the assumption that the items that were assessed and the test that was evaluated were exactly the same for the 2 levels of training; therefore, the calculation was performed with a pooled competent and expert level sample (n = 36).

To calculate the means of the CCI for the competent and expert levels, the competent sample was added to the expert sample (n = 36); the rubrics for the 3 tasks had excellent reliability (0.897 for the application of a tourniquet, 0.982 for the application of an emergency bandage and 0.805 for the application of haemostatic dressings).

## Discussion

The purpose of this study was to construct and validate a tool for the evaluation of responders in tactical casualty care simulations. The teaching activities carried out at EMISAN made it necessary to obtain valid instruments for evaluating training. Medical training is provided not only to military health personnel but also to health professionals in prehospital emergency medical services that provide tactical casualty care^(^ 18 ^)^.

No studies of these characteristics have been found for application in civilian prehospital care. Other published studies have validated evaluation instruments, for example, for simulations of external bleeding control by tourniquets in the military environment; however, this was done during the care under fire phase (threat to the responder and the patient), which differs from our study in that the simulations were performed during the threat-free phase (warm or cold zone according to the Hartford Consensus), which was not considered in the above studies^(^ 19 ^-^ 20 ^)^. The novelty of this study is the validation of the 3 tasks, which are fundamental in controlling bleeding both in the civilian and military environments.

It is important to train responders in these tasks because not being familiar with them implies they will not be used in situations where they could be of great utility, decreasing the chances of survival^(^ 21 ^)^. Valid evaluation instruments are necessary for the education of health professionals. As in our study, two-thirds of validation studies use internal consistency to validate assessment instruments^(^ 22 ^)^.

The results of our study show that the instrument presented is valid and reliable in the evaluation of simulations necessary for training regarding external bleeding control in incidents involving multiple victims. The internal consistency values were excellent (value of 1). Likewise, the reliability supports the use of the proposed rubric as an evaluation tool: the CCI results for each item are above the value considered good (0.4), except one (item 3 in the application of topical haemostats). These value can result from the items being developed for integration into simulations of different situations in which it is necessary to consider whether a tourniquet is necessary. The results for this item improved after increasing the sample and considering the competent and expert levels as a single sample. Likewise, the results for 2 other items (2 and 8) improved, as well as correlation index scores, when combining the samples. For 37% of the items, a correlation of 100% was obtained, most of which were found in the application of an emergency bandage simulation. This good result could be explained by the simplicity of the technique and the low degree of difficulty in its implementation.

We highlight the excellent values obtained for the average CCI for the 3 tasks (0.897, 0.982 and 0.805), which is why we consider that the evaluation instruments proposed are reliable for evaluating students being trained in the aforementioned tasks.

This work contributes to the development and validation of a rubric for the evaluation of tactical training in new scenarios, such as mass-casualty incidents, for prehospital care providers.

The results obtained are in accordance with other proposals^(^ 11 ^)^ that suggest that checklists during simulations can help to prioritize the sequence of the participant’s performance in simulated scenarios in which several tasks must be developed at the same time. The scenarios that are part of the simulation should include a number of components such as learning objectives, assessment tools, pre-screening, and debriefing, which are essential parts of the validation of assessment tools through peer review, clinical experts, evidence review and evaluation to establish usefulness of the simulation in the process of student training^(^ 23 ^)^.

The limitations of this study is sample size. The difficulty in obtaining an adequate sample size lies in the fact that, to date, the professionals who have used these instruments to control bleeding are in the Army. The geographical dispersion of these professionals after their completion of the courses in EMISAN provides limited opportunities to include these individuals in these studies. On the other hand, the unique experience of these professionals allows us to obtain reliable data for the study of an instrument for the evaluation of simulations in a threatening environment.

Future research should aim to develop evaluation instruments for simulations in which the environment exerts a great influence on the type of care: assessing safety, assessing the type of danger (explosions, firearms, etc.), determining necessary equipment, etc. Simulation training for this type of incident should recreate environments similar to those in which prehospital tactical care will be developed.

Validation is considered useful for both civilian and military training because these 3 techniques are used in the same way in both environments.

The authors continue the work to implement training in the general population. Validation of outcome assessment tools will help develop programmes that are useful and efficient. The Hartford Consensus recognizes the importance of providing this training to both health professionals and to non-health personnel who, because of their profession or activity, can become first responders.

## Conclusion

In conclusion, we emphasize that this study presents consistent results that support the use of our rubric for the evaluation of the application of tourniquets during threatening civilian environments for the evaluation of the application emergency bandages and haemostatic dressings under non-threatening conditions.

These assessment instruments are valid and reliable; therefore, their use is recommended to reliably evaluate training results from the simulations described above in both the military and civilian environments.
